# Hiatus Hernia: A Rare Cause of Acute Pancreatitis

**DOI:** 10.1155/2016/2531925

**Published:** 2016-03-15

**Authors:** Shruti Patel, Ghulamullah Shahzad, Mahreema Jawairia, Krishnaiyer Subramani, Prakash Viswanathan, Paul Mustacchia

**Affiliations:** ^1^Department of Internal Medicine, Nassau University Medical Center, East Meadow, NY 11554, USA; ^2^Department of Internal Medicine, Division of Gastroenterology and Hepatology, Nassau University Medical Center, East Meadow, NY 11554, USA

## Abstract

Hiatal hernia (HH) is the herniation of elements of the abdominal cavity through the esophageal hiatus of the diaphragm. A giant HH with pancreatic prolapse is very rare and its causing pancreatitis is an even more extraordinary condition. We describe a case of a 65-year-old man diagnosed with acute pancreatitis secondary to pancreatic herniation. In these cases, acute pancreatitis may be caused by the diaphragmatic crura impinging upon the pancreas and leading to repetitive trauma as it crosses the hernia; intermittent folding of the main pancreatic duct; ischemia associated with stretching at its vascular pedicle; or total pancreatic incarceration. Asymptomatic hernia may not require any treatment, while multiple studies have supported the recommendation of early elective repair as a safer route in symptomatic patients. In summary, though rare, pancreatic herniation should be considered as a cause of acute pancreatitis. A high index of suspicion for complications is warranted in cases like these.

## 1. Introduction

Hiatal hernia (HH) refers to herniation of elements of the abdominal cavity through the esophageal hiatus of the diaphragm.

The stomach is the most common organ to herniate. Other less common herniations include transverse colon, small intestine, and spleen. Herniation of the pancreas through the hiatus is an extremely rare occurrence. Pancreatic herniation leading to acute pancreatitis is an even more rare condition. In the world literature, from 1958 to 2011, 12 cases have been reported [1]. All reported patients were symptomatic except for one case reported by Katz et al. [2].

We describe a rare case of an overtly symptomatic patient diagnosed with acute pancreatitis who was found to have herniation of the stomach, the body, and the tail of the pancreas and the first part of the duodenum.

## 2. Case Presentation

A 65-year-old Caucasian male with no known past medical history came to the emergency room (ER) complaining of abdominal pain and vomiting for 1 day. He was in his usual state of health prior to an acute onset of epigastric pain which was dull and crampy in nature, 8/10 in intensity and associated with nausea and 10–12 episodes of nonbloody vomiting. He denied any other complaints. In the ER, his blood pressure (BP) was 165/91 mm of Hg, temperature (*T*) was 98 F, heart rate (HR) was 85/min, and the respiratory rate (RR) was 18. His abdomen was soft, with mild epigastric tenderness and normal bowel sounds. The rest of the physical exam was unremarkable.

**Figure 1:**
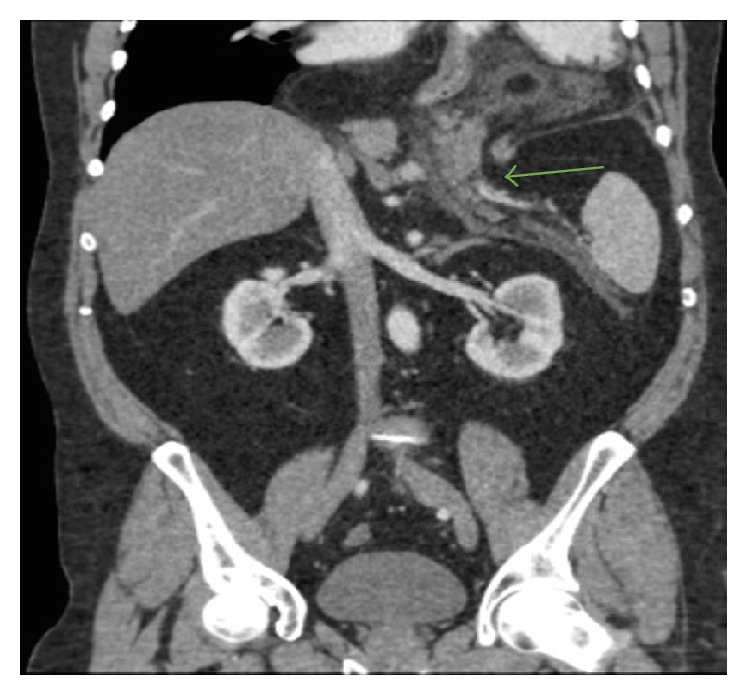
Coronal plane; hiatal hernia; herniation of the stomach, the body and tail of the pancreas, and the first part of the duodenum.

**Figure 2:**
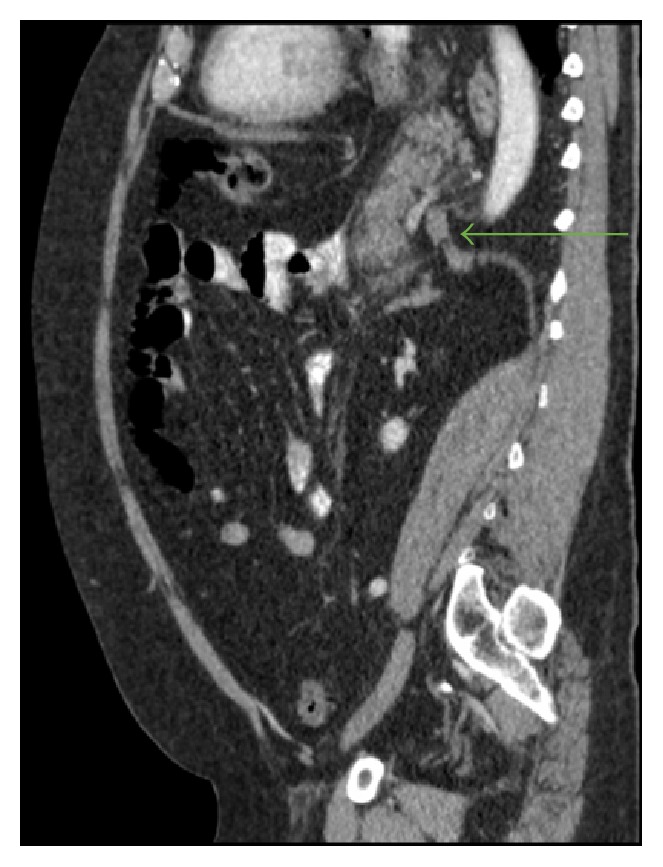
Sagittal plane; diaphragmatic crura impinging the pancreas.

His initial blood work revealed a lipase of 2950. Computed tomography (CT) of abdomen/pelvis with radio contrast (Figures 1 and 2) was done which showed a large hiatal hernia containing the entire stomach, the first part of the duodenum, and most of the body and the tail of the pancreas. This was associated with peripancreatic inflammation highly suspicious for acute pancreatitis.

The patient underwent an esophagogastroduodenoscopy (EGD) on day 4 of admission which revealed erythematous mucosa of the fundus and body of the stomach, Barrett's esophagus, and a sliding HH. Biopsy revealed squamocolumnar mucosa with mild chronic inactive inflammation and no helicobacter pylori identified. The patient refused any surgical intervention. He clinically improved and was discharged with outpatient follow-up.

## 3. Discussion

HH corresponds to the transient or permanent migration of the stomach through the diaphragm into the chest which may be accompanied by other abdominal organs as in this case. The most common organs to accompany the stomach are the colon (usually the splenic flexure), loops of small bowel, and omentum. A large HH is a hernia that includes at least 30% of the stomach in the chest. Many reports of giant HH have been published, but a giant HH with pancreatic prolapse is extremely rare [3]. The head of the pancreas and the duodenum are typically held down by the ligament of Treitz and hence herniation of the pancreas usually does not occur [4]. It is proposed that stretching of the transverse mesocolon leads to increased laxity of the posterior adhering fascia thereby mobilizing the pancreas, hence causing herniation. Overall, herniation of the pancreas is extraordinary with only 12 cases reported until now.

HH are more common in Western countries [5]. Burkitt and James suggest that the Western, fiber-depleted diet leads to a state of chronic constipation and straining during bowel movement, which could explain the higher incidence of this condition in Western countries. Hiatal hernias are more common in women than in men. The frequency of HH increases with age, from 10% in patients younger than 40 years to 70% in patients older than 70 years.

HH is classified into two types depending on the position of the gastroesophageal (GE) junction and the extent of herniated stomach: sliding hernias and paraesophageal hernias [6, 7]. Type I, also called sliding hernias, occurs when the GE junction migrates through the hiatus into the posterior mediastinum. It is the most common type, accounting for 85–95% of all the cases [8, 9]. Types II, III, and IV, also called paraesophageal hernias, are less common and account for the remaining 5–15% of the cases [10, 11]. Type II occurs when the GE junction is in its normal position and the fundus herniates through the hiatus along its side. Type III is a combination of types I and II wherein there is a protrusion of the stomach through the hiatus along with a displaced GE junction. Type IV is defined by herniation of the stomach with other organs into the chest.

HH may be asymptomatic or patients might complain of heartburn, belching, dysphagia, abdominal pain, nausea, chest pain, or cough. In most patients, the cause of the HH is unknown. Some people are born with a weakness or an especially large hiatus. It is believed that increased pressure in the abdomen from chronic coughing, straining during bowel movements, pregnancy and delivery, obesity, and abdominal ascites may contribute to the development of the HH. Rarely, iatrogenic or traumatic diaphragmatic hernia (DH) may occur. They account for less than 1% of all the DH [12]. Iatrogenic hernias can occur due to alterations in the normal anatomy from surgical dissection of the hiatus. This may be due to disruption of a previous hiatal closure, postoperative gastric dilatation, disruption of the phrenoesophageal membrane by operative dissection, and failure to recognize esophageal shortening or an existing hiatal defect [13]. Etiologies of iatrogenic diaphragmatic defects include Ivor Lewis procedure, antireflux procedures, esophagomyotomy, partial gastrectomy, gastric banding procedures, and misguided chest tubes or thoracoabdominal incisions in which the diaphragm is taken down [14].

HH can be complicated by intermittent bleeding episodes from associated esophagitis, erosions, and esophageal ulcers; iron deficiency anemia; incarceration; strangulation and perforation. Acute pancreatitis complicating a diaphragmatic hernia is rare and multiple theories have been proposed to explain it. Acute pancreatitis occurring in the case of a pancreatic hernia may be caused by repetitive trauma as it crosses the hernia, ischemia associated with stretching at its vascular pedicle, [15, 16], or intermittent folding of the main pancreatic duct. Total incarceration of the pancreas may also contribute to pancreatitis [17].

Many diagnostic modalities are available including plain chest X-ray film, barium upper gastrointestinal series, CT imaging, and EGD. Asymptomatic hernia might not require any treatment. HH with gastroesophageal reflux disease can be managed medically. Symptomatic HH (e.g., chest pain and dysphagia), HH with severe esophagitis, and types II, III, and IV HH might require surgical intervention which includes reduction of the hernia, closure of the hiatal defect, and antireflux procedure. Hiatal hernias can be repaired by a transabdominal or transthoracic approach [18]. Laparoscopic hiatal hernia repair and open transabdominal repair are equally effective. Laparoscopic repair is the preferred approach for the majority of the hiatal hernias as it is associated with reduced rate of perioperative morbidity and mortality and shorter hospital stays [19]. The operative principles of a hernia repair are the same for laparoscopic and open approaches. They involve the dissection of the sac and reduction of the hernia followed by complete excision of the sac from the mediastinum. After the esophagus is mobilized, the hiatal defect is closed by interrupted nonabsorbable sutures (primary repair) if the defect is small or using a mesh for the larger defects. Gastric fixation, such as anterior gastropexy may safely be used in addition to hiatal repair to prevent recurrence.

The risk of these hernias becoming incarcerated, leading to strangulation or perforation, is approximately 5%. Elective repair often is advised in symptomatic patients. In a study by Sihvo et al. [20], of the 563 patients in the surgical group, the overall mortality was 2.7% whereas mortality of the 67 patients in the conservative treatment group was 16.4%. 13% of the deaths might have been avoided with elective surgical intervention. Of the 32 patients who died, over half had type III or type IV hiatal hernias; 4 patients had type II hiatal hernias, with the remaining 3 deceased having an unknown type. In a Swiss study [21], of the 354 laparoscopic paraesophageal hernia repairs, age at 70 years or older was significantly associated with postoperative morbidity (24.4%) and mortality (2.4%) relative to those younger than 70 years. These findings support early elective repair as a safer route in symptomatic patients.

Pancreatitis caused in our patient was treated with fluids, analgesia, antiemetics, and gradual advancement of diet as tolerated by the patient. Our patient did not undergo surgery since the patient only wanted conservative management at that time.

## 4. Conclusion

Pancreatic herniation is a rare entity which may lead to even rarer complications of pancreatitis. Symptomatic herniation is best treated with surgery. Our patient's large hernia was not surgically repaired, despite our recommendations and an explanation of the potentially fatal risk. Elective surgery is found to be safer than emergent surgery in a patient that has suffered a serious complication.
